# Ocular circulation change in optic disc melanocytoma – a case report and a review of the literature

**DOI:** 10.1186/s12886-023-02785-9

**Published:** 2023-01-23

**Authors:** Tsung-Ying Tsai, Yueh-Ju Tsai, Yen-Chang Chu, Yih-Shiou Hwang, Yi-Lin Liao

**Affiliations:** 1 Department of Ophthalmology, Chang Gung Memorial Hospital, Linkou, Taiwan; 2 Department of Medicine, College of Medicine, Chang Gung University, Taoyuan, Taiwan

**Keywords:** Optic disc melanocytoma, Ocular circulation, Optical coherence tomography angiography, Laser speckle flowgraphy, Case report

## Abstract

**Background:**

Ocular circulation in optic disc melanocytoma (ODM) has not yet been well evaluated. We quantitatively evaluated longitudinal changes in the morphology and circulation hemodynamics of the disc and macula using optical coherence tomography angiography (OCTA) and laser speckle flowgraphy (LSFG) in a patient with optic disc melanocytoma.

**Case presentation:**

A 50-year-old woman was referred to our hospital due to a dark pigmented tumor over the superior optic disc area of the left eye noted on physical examination. At the first visit, the patient’s best-corrected visual acuity (BCVA) was 20/20 in both eyes, and the intraocular pressure (IOP) was 17 and 18 mmHg in the left and right eyes, respectively. Fluorescein angiography (FA) showed blockage of fluorescence in the topography of the lesion, and indocyanine green angiography (ICGA) showed hypofluorescence at all times. On LSFG, a low mean blur rate (MBR) was noted in the optic disc all area (MBRa) and tissue (MBRt) compared to the contralateral eye at the first visit and at the 3-month follow-up. A relatively low MBR was also detected in the macular area of the affected eye and the tumor itself. OCTA detected blood vessel networks in the deep retinal layer of the tumor. The visual field showed no specific defects. During follow-up, there was no tumor enlargement or vision decrease.

**Conclusions:**

We found that a lower MBR of the disc and macula area was noted on LSFG in this patient with optic disc melanocytoma, and it was continually observed at the 3-month follow-up. Although blood vessel networks in the deep retinal layer of the tumor were detected by OCTA, vascular compromise in the surrounding disc area and macula was found. Therefore, these results further increase our knowledge about the role that circulation impairment plays in the pathogenesis of the disease while vision is unaffected.

**Supplementary Information:**

The online version contains supplementary material available at 10.1186/s12886-023-02785-9.

## Background

Optic disc melanocytoma (ODM) is a rare, benign and deeply pigmented tumor that usually arises from the optic nerve head, sometimes involving the retina (30%) and choroid (54%) [1–3]. ODM is typically asymptomatic but could have a 1 to 2% probability of malignant transformation to melanoma [4, 5]. Most tumors occur unilaterally with a slight female predominance and location inferior to the optic disc [1–3].

A total of 10–19% of lesions grow over several years after diagnosis, reaching 32% at the 10-year follow-up [1, 2, 6]. Although tumors grow slowly, and symptoms are not common, the patient can have visual deterioration and visual field defects [7–9]. Therefore, it is necessary to continue observation.

Multiple modalities, such as optical coherence tomography (OCT), fundus autofluorescence (FAF), fluorescein angiography (FA) and sonography, have been developed and have enabled ophthalmologists to evaluate tumors on diagnosis and follow-up [1, 2, 10–12]. In recent years, OCT angiography (OCTA) has served as a noninvasive and promising technique for evaluating the microcirculation, providing vascular areas and densities [12–17].

Laser speckle flowgraphy (LSFG) has been used to quantitatively investigate ocular blood flow velocity, targeting moving red blood cells with a diode laser (wavelength, 830 nm) to illuminate the ocular fundus [18]. Mean blur rate (MBR) calculation in the blood vessels and tissues (capillaries) of the entire optic nerve head and macula has demonstrated good correlation with capillary blood flow and good reproducibility [19–21]. Therefore, LSFG is a suitable modality for monitoring hemodynamic changes in the ocular circulation during the course of various chorioretinal diseases [20].

In this study, we aimed to use LSFG and OCTA for morphological and circulatory evaluations of the disc and macula in a patient with ODM. Longitudinal changes could also be observed on the follow-up exam.

## Case presentation

A 50-year-old woman was referred to our hospital due to a dark pigmented tumor over the superior optic disc area of the left eye noted from a recent physical exam. She denied any visual symptoms. The patients’ medical and family histories were unremarkable.

**Fig. 1:**
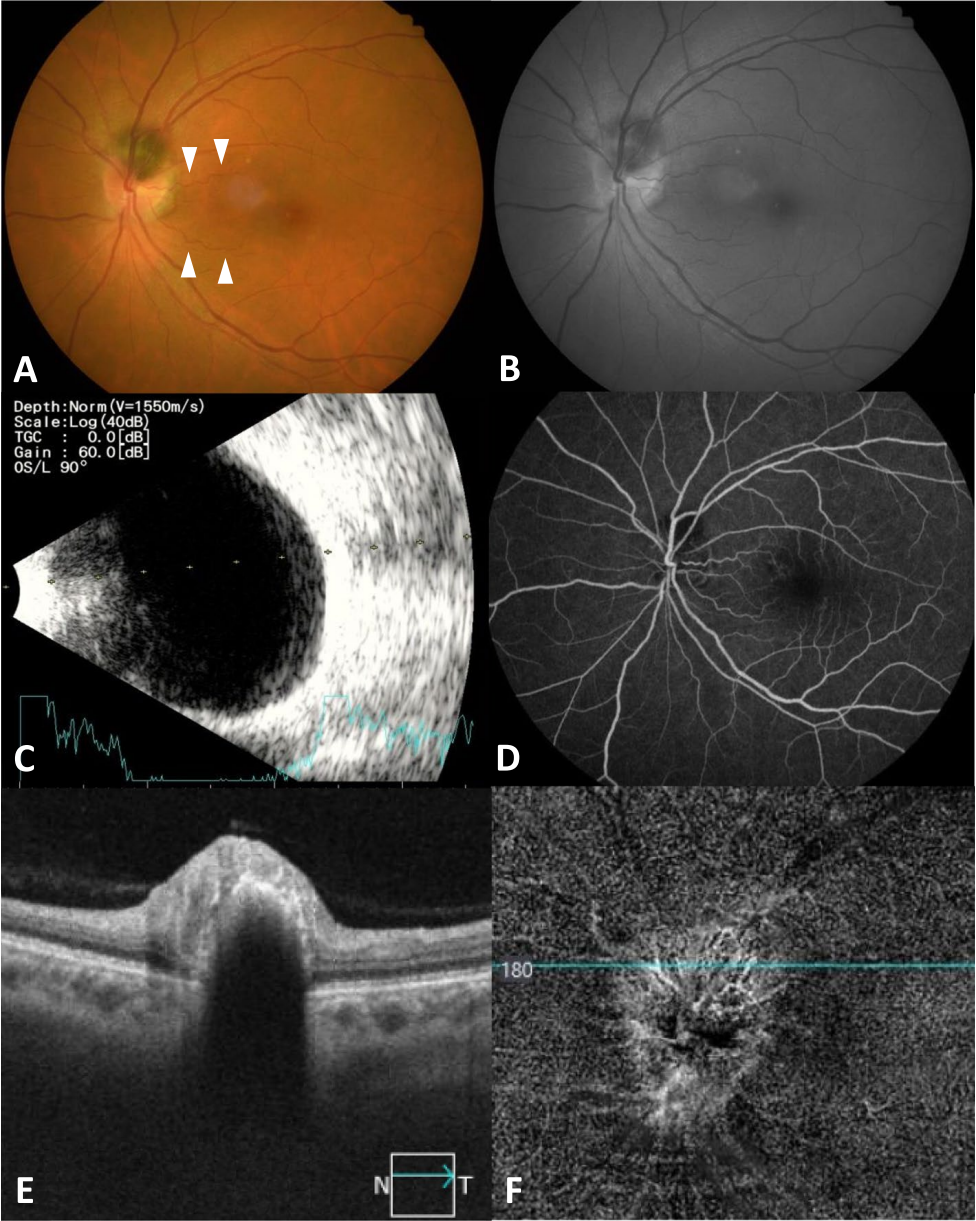
Photographs of the left eye at the initial visit with a patient with optic disc melanocytoma. **A** Fundus photography showed a dome-shaped lesion with dark pigmentation extending from the optic disc to the superior side of the optic nerve head. Tortuous cilioretinal arteries are also noted as arrowheads. **B** FAF revealed hypoautofluorescence corresponding to the pigmented masses and adjacent retina. **C** B-scan ultrasonography showed no detection of tumor lesions. **D** FA showed blockage of fluorescence in the topography of the lesion and prominent cilioretinal arteries. **E** SD-OCT showed a dome-shaped mass with a brightly reflective anterior surface (green arrowheads) and a posterior optical shadow (white arrowhead). **F** OCTA detected blood vessel networks in the deep retinal layer of the tumor

**Fig. 2:**
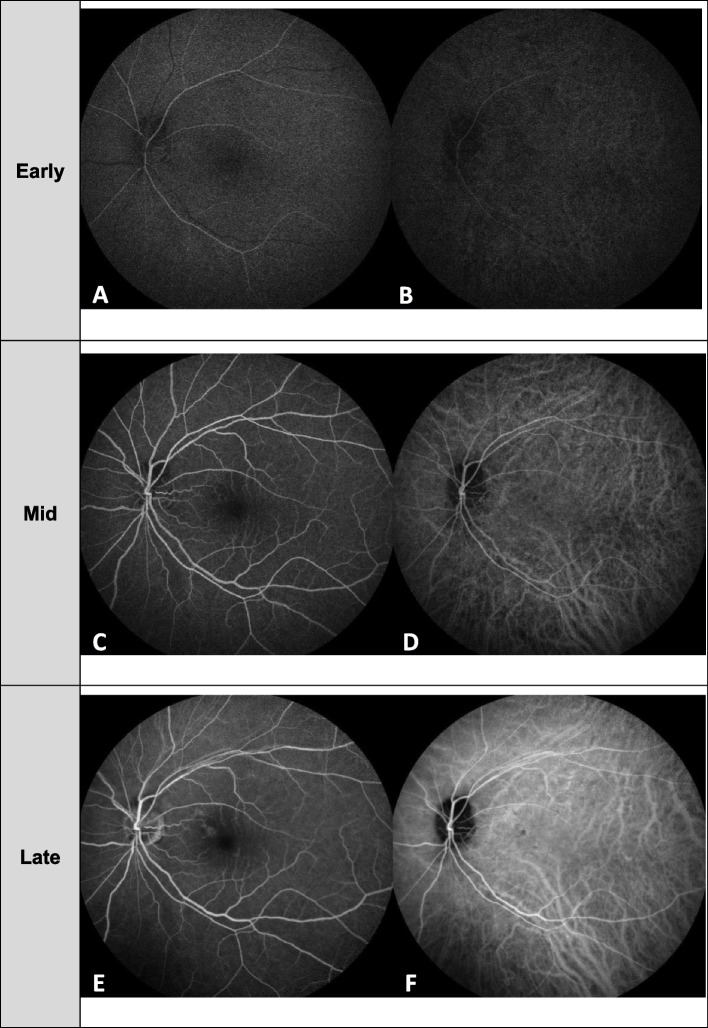
Serial change of retinal and choroidal vasculature in FA/ICG images. From early phase of FA (**A**) and ICG (**B**), mid phase of FA (**C**) and ICG (**D**) to late phase of FA (**E**) and ICG (**F**). **A**,** C**,** E**. FA showed blockage of fluorescence in the topography of the lesion without any dye leakage or vessel density. **B**,** D**,** F**. ICGA showed hypofluorescence from the early (**B**) to the late phase (**F**) corresponding to the lesion. Notably, prominent cilioretinal arteries appeared to originate from the retinal vasculature and showed mild leakage at vessel end near macula region of FA (**E**)

**Fig. 3:**
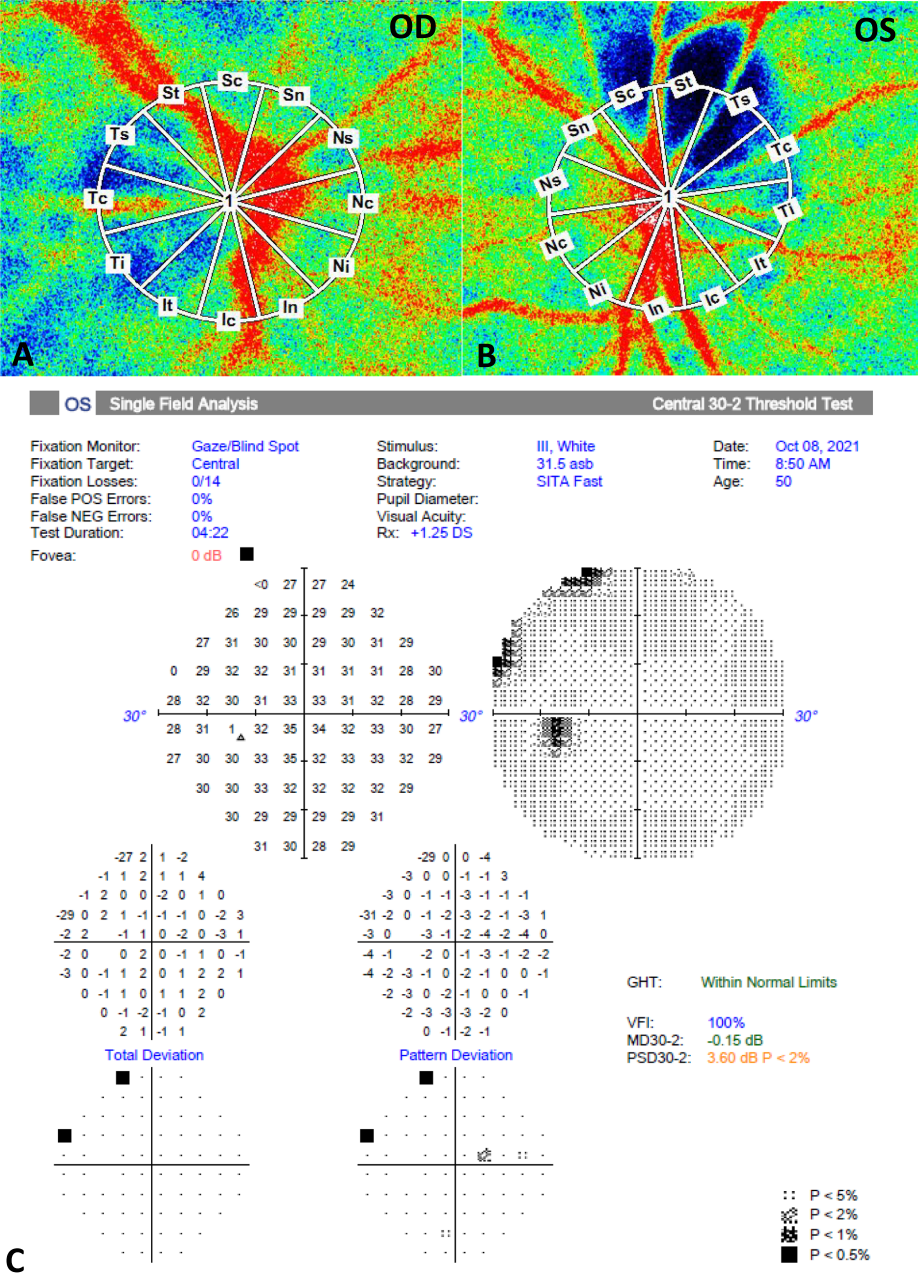
**A**,** B** The color map of LSFG demonstrated markedly reduced blood flow in the tumor and adjacent parapapillary region of the left eye (**B**) compared to the right eye (**A**) at the initial visit. The measurement circles shown here were calculated for the MBR. **C** HVF central 30–2 threshold test showed no specific visual field defect at the initial visit

Initial examination revealed visual acuity in both eyes of 20/20, and the intraocular pressure was 17 mmHg in the right eye and 18 mmHg in the left. Slit-lamp examination revealed no abnormal findings in the anterior segment and lens in both eyes. Funduscopy showed a dome-shaped, pigmented lesion extending from the optic disc to the superior pole in the left eye. The macular area showed no edema but tortuous cilioretinal arteries (Fig. 1A). On ocular ultrasound, no detection of tumor lesions or invasion was noted for OS (Fig. 1C). FA showed blockage of fluorescence in the topography of the lesion without any dye leakage or vessel density throughout all phases (Figs. 1D and 2A, C, E). Indocyanine green angiography (ICGA) showed hypofluorescence from the initial to the late phase within the lesion (Fig. 2B, D, F). Spectral-domain optical coherence tomography (SD-OCT; Spectralis; Heidelberg. Engineering, Heidelberg, Germany) imaging showed a dome-shaped mass with a brightly reflective anterior surface and posterior optical shadow (Fig. 1E). Blood vessel networks were observed in the tumor region on OCTA (PLEX Elite 9000; Carl Zeiss Meditec, Inc., Dublin, USA) and were most prominent in the deep retina layer (Fig. 1F). Intact blood vessel networks were observed in the macular region. For potential effects on the visual field, the Humphrey visual field (HVF) central 30–2 threshold test showed no specific visual field defect’s (Fig. 3C). The patient was diagnosed with optic disc melanocytoma OS and was followed up with no treatment. Three months later, her BCVA remained at 20/20, and her IOP was 19 mmHg.

To quantitatively examine ocular blood flow, LSFG measurements using LSFG-RetFlow (NIDEK Co., Ltd., Gamagori, Aichi, Japan) were obtained at the first visit and 3-month follow-up visit. The optic disc and macular region were measured five consecutive times in the affected eye and the fellow eye. The pupils were dilated with 1.0% tropicamide and 10% phenylephrine 30 minutes prior to the exam. During each testing period, eye movement and focus adjustment were monitored by an experienced technician using real-time captured images. To evaluate the blood flow velocity, the measurement circles were manually managed according to color fundus photographs and LSFG color maps. The MBR, a quantitative index of relative blood flow velocity, was calculated in each circle using LSFG Analyzer software (version 3.0.47; Softcare). During follow-up, each circle was automatically set by the software at the same site as the previous baseline measurement.

A previous study revealed a bilinear relationship between choroidal blood flow and ocular mean perfusion pressure (OPP) in healthy eyes [22]. To evaluate the possible influence of such a physiological response on our results, we calculated the patient’s blood pressure and IOP to obtain OPP. As defined, the mean blood pressure was calculated by the following equation: BPm = BPd + 1 / 3(BPs - BPd). (BPm: mean blood pressure, BPs: systolic blood pressure, BPd: diastolic blood pressure). OPP was calculated using the following equation: OPP = 2 / 3 BPm - IOP.

**Fig. 4:**
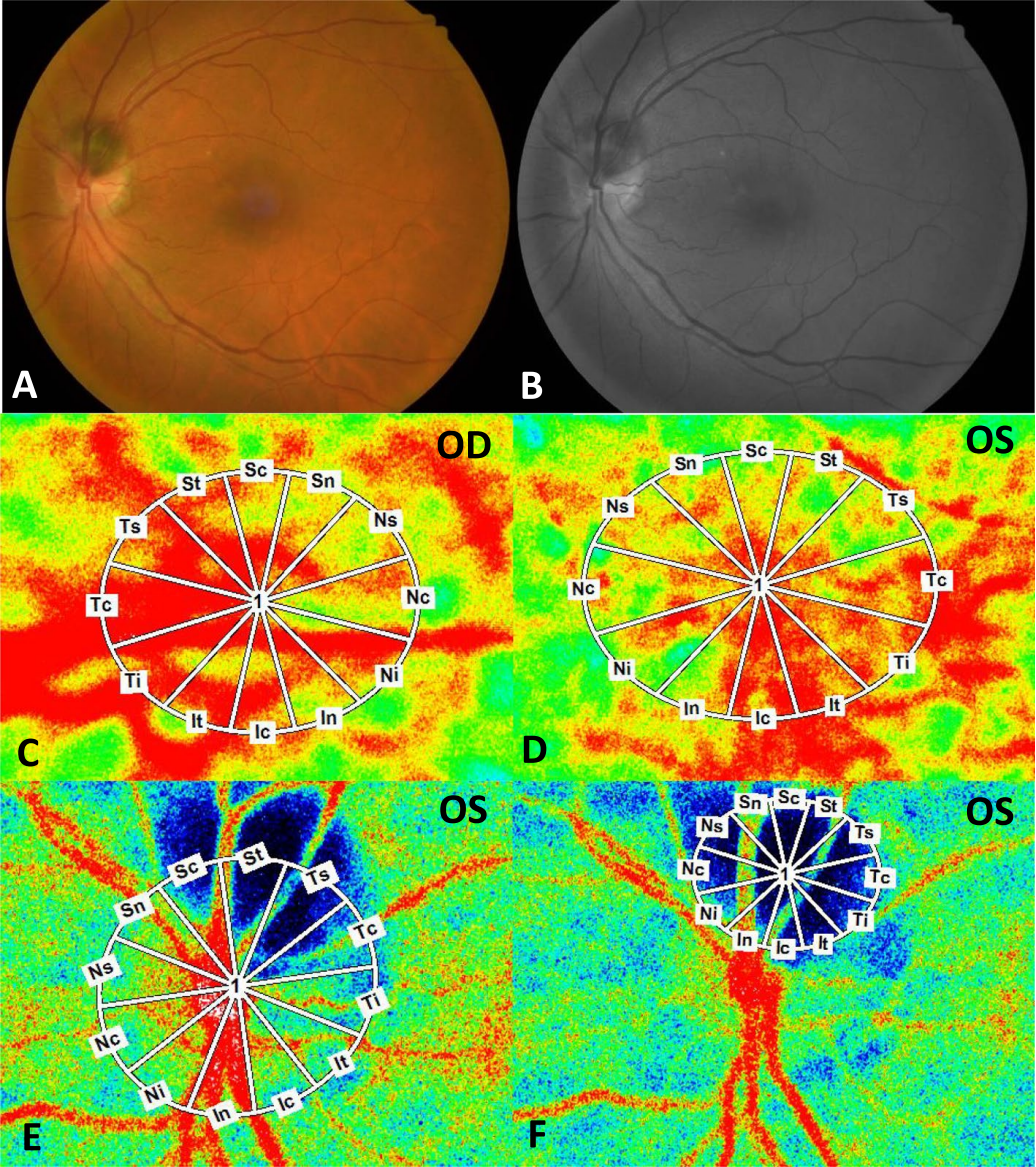
Photographs of the left eye at the 3-month follow-up in a patient with optic disc melanocytoma. **A**,** B** Fundus photography and FAF showed relatively stable size and other characteristics of the tumor in the left eye compared to the initial visit. **C**,** D** The color maps of LSFG from each eye are shown. Measurement circles were set at the macula area (fovea as center) and calculated for MBR using the same method as the optic disc area. **E** At the 3-month follow-up, the circle was automatically set at the same site as the previous baseline. **F** The color map of the tumor and circled area along the border of the tumor are presented for measurement

In the affected eye, LSFG demonstrated markedly reduced blood flow in the tumor and adjacent parapapillary region at the initial (Fig. 3B) and 3-month follow-up visits (Fig. 4E). A lower MBR was noted in the optic disc all area (MBRa) and tissue (MBRt) compared to the contralateral eye (MBRa: 23.0 ± 0.8 vs. 26.5 ± 1.9, OS vs. OD; MBRt: 12.7 ± 0.5 vs. 15.3 ± 1.1, OS vs. OD) at the initial visit (Table 1). Three months later, slight decreases in MBRa (16.9 ± 0.4 vs. 21.7 ± 0.4, OS vs. OD) and MBRt (10.5 ± 0.2 vs. 14.0 ± 0.2, OS vs. OD) were detected. The rate of change in the MBRa was 26.5% in the left eye and 18.1% in the right eye, while the MBRt was 17.3% in the left eye and 8.5% in the right eye. A relatively low MBR (23.6 ± 0.3 vs. 27.2 ± 1.4; 18.8 ± 0.2 vs. 22.3 ± 1.1; 0 vs. 3 M, OS vs. OD) was also detected in the macular area of the affected eye (Fig. 4D) and the tumor itself (MBR = 5.8 ± 0.2) (Fig. 4F).

**Table 1:** Measurement of MBR in both eyes by LSFG software in the optic disc and macula area

	Optic disc area				Macula area	
	MBRa		MBRt		MBR	
	0	3 M	0	3 M	0	3 M
**OD**						
Mean	26.5	21.7	15.3	14.0	27.2	23.3
SD	1.9	0.4	1.1	0.2	1.4	1.1
**OS**						
Mean	23.0	16.9	12.7	10.5	23.6	18.8
SD	0.8	0.4	0.5	0.2	0.3	0.2

0 = initial visit; 3 M = follow-up at 3 months

Five consecutive measurements are presented as the mean and standard deviation

*MBR* Mean blur rate, *MBRa* Mean blur rate in optic disc all area, *MBRt* Mean blur rate in tissue area, *SD* Standard deviation

The measured OPP was 57.8 and 61 mmHg in the affected eye at the initial visit and 3-month follow-up, respectively. In the contralateral eye, the measured OPP was 58.8 mmHg at the initial visit and 61 mmHg at 3 months. These measurements were comparable without obvious alterations in values.

## Discussion and conclusions

In the present study, we quantitatively evaluated the ocular blood flow velocity and vascular structures in the optic disc and macular region using LSFG and OCTA in a patient with a diagnosis of optic disc melanocytoma.

Vascularization of ODM is an important marker to evaluate tumor growth. Lee et al. reported that vascularization seemed to predict tumor growth on FA [23]. Shields et al. called this vascularization “intrinsic vascularization” in their study, and they also found it significantly correlated with tumor enlargement [6]. However, it could have limitations for demonstrating such findings on FA due to the low signal intensity and heavy pigmentation of the tumor, which would block the penetration of light, making it difficult to evaluate the vasculature, as in our case. In the late phase, FA could possibly be influenced by leakage or staining. In contrast, OCTA can overcome the aforementioned obstacles since it is not influenced by either pigmentation or leakage of dye, and it provides better visualization of the vasculature [12, 14].

OCTA has recently been proposed as a noninvasive imaging modality in the evaluation of vascular structures in chorioretinal tumors. Cennamo et al. presented two cases of ODM in which OCTA revealed a bloodstream defect in the deep retinal layer and suspected in the choriocapillaris layer [24]. Other studies have also reported an abnormal blood vessel network in the deep retinal layer [13], both the superficial and deep retinal layers [14], and dense blood vessels in the tumor [12, 15]. In our study, our patient showed blood vessel networks in the tumor region that were most prominent in the deep retinal layer, consistent with previous reports. Moreover, ODM eyes could have a decrease in optic nerve vascularization in the radial peripapillary capillary plexus (3 of 5 eyes) and the outer retinal plexus (4 of 5 eyes) [16]. Our case further disclosed that tortuous cilioretinal arteries were observed in the ODM eye with corresponding findings on OCTA, indicating that the tumor could affect the outflow of major vessels from the peripapillary area. We also evaluated the macular region with OCTA to determine whether any association with the vascularization of the macula existed. Burgos-Blasco et al. found that two patients who presented with tumor growth had less vascularization in the deep plexus in the ODM eye than in the fellow eye, and such a finding could become a predictive test for tumor growth risk [16]. Although our case did not show alterations in the macular vascularization of the affected eye, a relatively low MBR was detected in the macular area on LSFG. This finding provides us with more information about the potential effect of the optic nerve lesion on the macular circulation, and LSFG could be an effective evaluation tool prior to vascular structural changes.

In the affected eye, a lower MBR was detected at the optic disc region and macular region at the initial visit, and it was continually observed 3 months later. Although a decreasing trend in MBR was observed in the contralateral eye at 3 months compared to the initial visit, it was relatively unaffected and was higher than that in the affected eye over the course of observation. Since the measured OPP (see Additional file 1) was comparable without obvious alterations during the follow-up, the MBR changes in this patient could be concluded to be due to choroidal blood flow velocity but not systemic hemodynamics. These results suggest that, although blood vessel networks were present, circulation disorders developed in the ODM tissues.

In the present study, we confirmed the morphological and circulatory patterns of ODM via OCTA and LSFG, which could overcome the limitations of FA and ICGA. LSFG is a suitable modality to monitor hemodynamic changes in the ocular circulation in ODM prior to those morphological changes undetected by other imaging studies. In our ODM case, a functional compromise of circulation in the surrounding disc and even the macula area was found on LSFG. Although the patient has not yet suffered from visual field changes, it is worth evaluating the peripapillary and macular perfusion patterns and correlating them with the prognosis of the patient in longer-term follow-up. Further studies with a larger number of cases are needed to establish the usefulness of the MBR in LSFG in this disease.

## Supplementary Information

**Additional file 1. **Intraocular pressure, systemic blood pressure and heart rate. Measured OPP was comparable without obvious alteration during the follow-up.

**Additional file 2. **Regional MBR in the right (A) and the left eye (B) of optic disc. Corresponding decrease MBR in vascular regions (Sn, Sc and St) of the left eye, while MBR of other vascular regions showed intact and were comparable to the right eye.

## Data Availability

All data generated or analyzed during this study are included in this published article.

## Abbreviations

OCTA: Optical coherence tomography angiography
LSFG: Laser speckle flowgraphy
BCVA: Best-corrected visual acuity
IOP: Intraocular pressure
FA: Fluorescein angiography
ICGA: Indocyanine green angiography showing hypofluorescence
MBR: Mean blur rate
MBRa: Mean blur rate in the optic disc all area
MBRt: Mean blur rate in the tissue area
ODM: Optic disc melanocytoma
OCT: Optical coherence tomography
FAF: Fundus autofluorescence
SD-OCT: Spectral-domain optical coherence
HVF: Humphrey visual field
OPP: Ocular perfusion pressure
BPm: Mean blood pressure
BPs: Systolic blood pressure
BPd: Diastolic blood pressure
