# Supplementary material for: Ocular circulation change in optic disc melanocytoma – a case report and a review of the literature
Source: BMC Ophthalmol. 2023 Jan 23;23:33. doi: 10.1186/s12886-023-02785-9 (PMC9869588; doi:10.1186/s12886-023-02785-9)
Supplement: Supplementary file 1 — Additional file 1. Intraocular pressure, systemic blood pressure and heart rate. Measured OPP was comparable without obvious alteration during the follow-up. [file 12886_2023_2785_MOESM1_ESM.docx]

**Additional File 1.** Intraocular pressure, systemic blood pressure and heart rate.

|  | **0** | | **3M** | |
| --- | --- | --- | --- | --- |
|  | **OD** | **OS** | **OD** | **OS** |
| **IOP** | 17 | 18 | 19 | 19 |
| **MBP** | 113.7 | 113.7 | 120 | 120 |
| **MOPP** | 58.8 | 57.8 | 61 | 61 |
| **HR** | 69 | 69 | 72 | 72 |

0 = initial visit; 3M= follow-up at 3 month.

IOP: intraocular pressure; MBP: mean blood pressure; MOPP: Measured ocular perfusion pressure; HR: heart rate.
