# Supplementary material for: Ocular circulation change in optic disc melanocytoma – a case report and a review of the literature
Source: BMC Ophthalmol. 2023 Jan 23;23:33. doi: 10.1186/s12886-023-02785-9 (PMC9869588; doi:10.1186/s12886-023-02785-9)
Supplement: Supplementary file 2 — Additional file 2. Regional MBR in the right (A) and the left eye (B) of optic disc. Corresponding decrease MBR in vascular regions (Sn, Sc and St) of the left eye, while MBR of other vascular regions showed intact and were comparable to the right eye. [file 12886_2023_2785_MOESM2_ESM.docx]

**Additional File 2. Regional MBR in the right (A) and the left eye (B) of optic disc.**

**A**

**
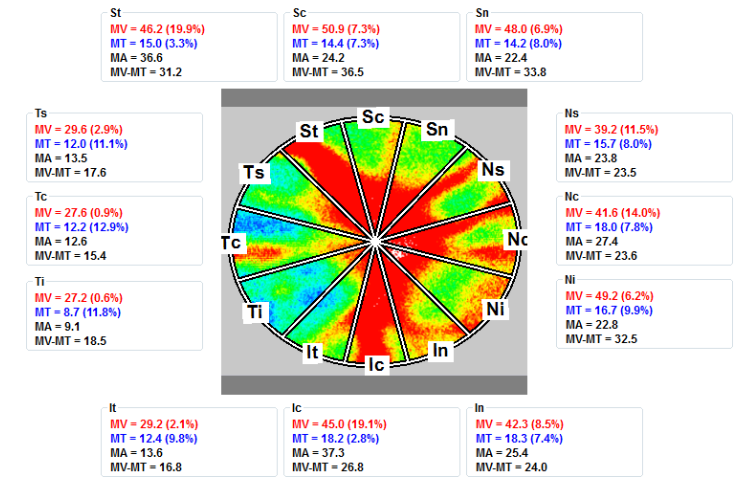
**

**B**


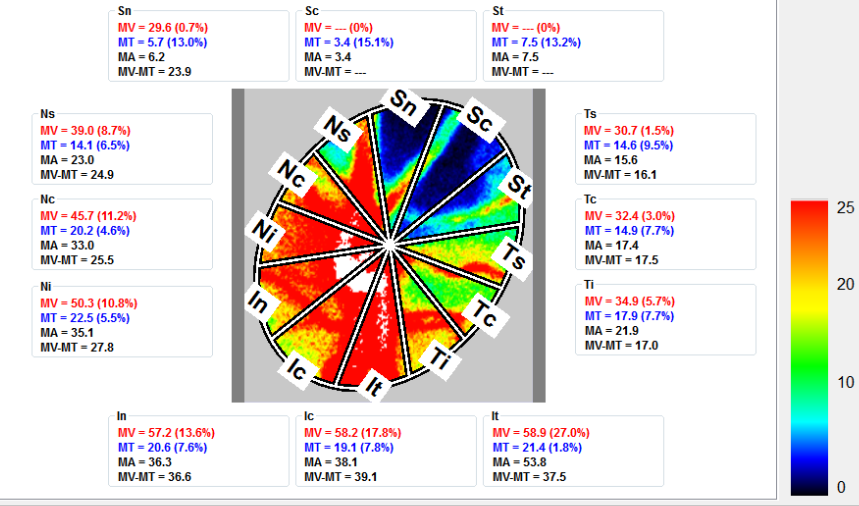


MBR: Mean blur rate; MV: Mean blur rate in vascular area; MT: Mean blur rate in tissue area; MA: Mean blur rate in optic disc all area.
